# Mechanistic Insights into Autoinhibition of the Human Flippase ATP8B1

**DOI:** 10.1007/s00232-026-00391-6

**Published:** 2026-07-28

**Authors:** Michelle Juknaviciute Laursen, Mathilde Roth, Poul Nissen, Charlott Stock, Thibaud Dieudonné

**Affiliations:** 1 DANDRITE, Nordic EMBL Partnership for Molecular Medicine, Department of Molecular Biology and Genetics, Aarhus University, Aarhus, Denmark; 2 Institute for Integrative Biology of the Cell (I2BC), CEA, CNRS, Université Paris-Saclay, 91198 Gif-sur-Yvette, France

**Keywords:** P-type ATPase, Flippases, P4-ATPase, Autoinhibition, ATP8B1, PFIC1, Intrahepatic cholestasis

## Abstract

P4-ATPases are lipid flippases that maintain membrane phospholipid asymmetry by transporting specific phospholipids from the exoplasmic to the cytosolic leaflet, an essential process for membrane integrity, trafficking and signaling. Several P4-ATPases are tightly regulated by autoinhibitory N- and C-terminal extensions, yet the molecular basis of this regulation remains incompletely understood. Here, we investigated the autoinhibition mechanism of the human flippase ATP8B1 using trans-inhibition assays with synthetic peptides derived from its C-terminal tail. Using purified C-terminally truncated ATP8B1-CDC50A, we systematically assessed the inhibitory properties of peptides corresponding to distinct segments of the C-terminus. We show that the distal disordered region of the C-terminal tail significantly contributes to autoinhibition, likely through transient interactions with the cytosolic domains. We further identify a critical interaction between R1228 in the C-terminal tail and E219 in the A-domain, whose disruption markedly reduces inhibitory potency. In addition, we demonstrate that a minimal peptide spanning residues 1216-1228, which bridges the A- and N-domains in the autoinhibited conformation, is sufficient to inhibit ATPase activity. Together, these results refine the molecular description of ATP8B1 autoinhibition, open the way for structure-based activation strategies and provide insight into conserved regulatory mechanisms among P4-ATPases.

**Graphical Abstract:**

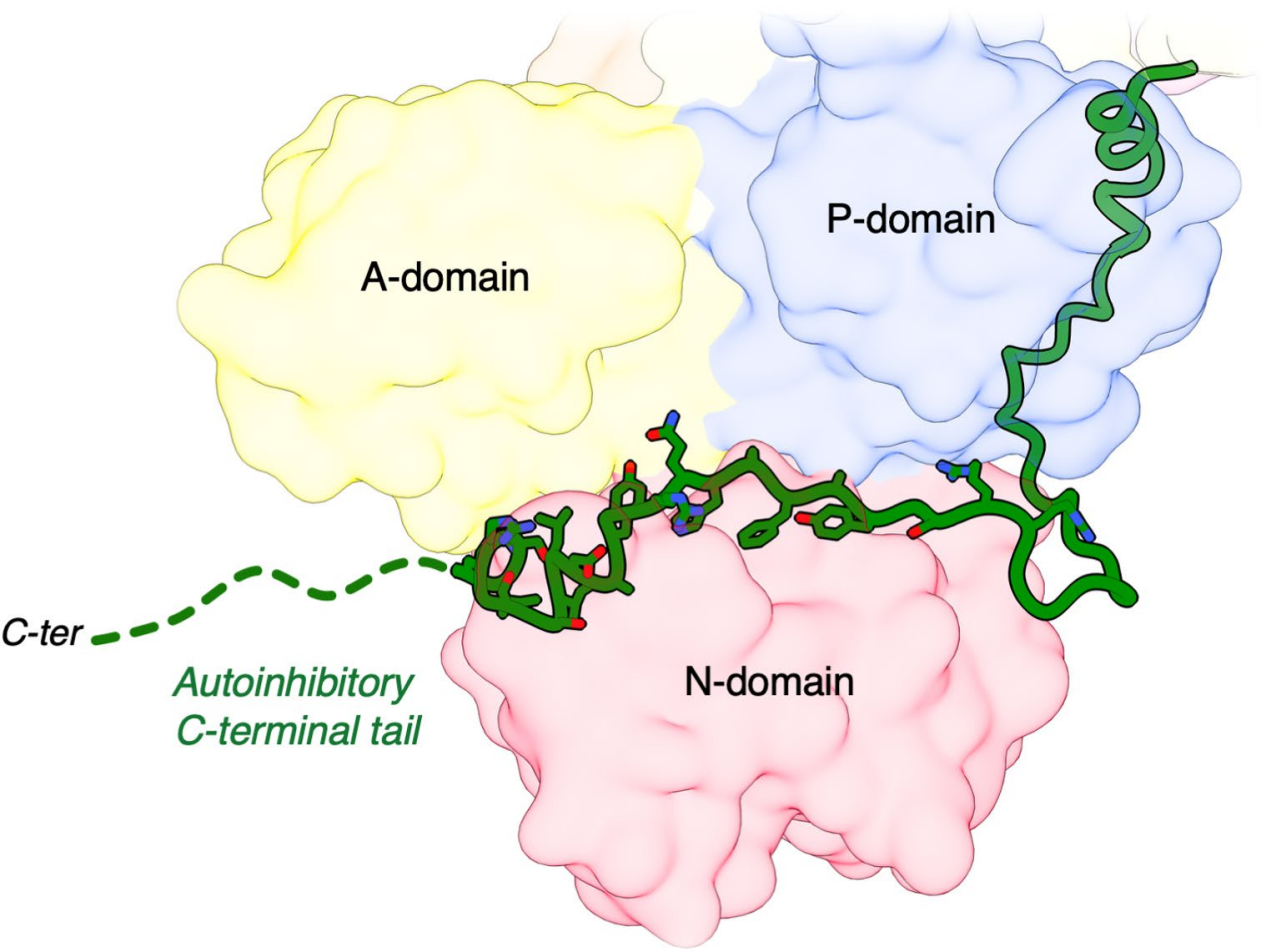

**Supplementary Information:**

The online version contains supplementary material available at 10.1007/s00232-026-00391-6.

## Introduction

Membranes of the late secretory pathway of eukaryotes are characterized by an asymmetric distribution of phospholipids. This asymmetry is mainly defined by an enrichment of sphingolipids and phosphatidylcholine (PtdCho) in the exoplasmic leaflet, whereas phosphatidylserine (PtdSer) and phosphatidylethanolamine (PtdEth) are mostly found in the cytosolic leaflet (Caputo et al., 2025). This asymmetry is essential for numerous cellular processes, in particular signaling and membrane trafficking events, where the enrichment of PtdSer on the cytoplasmic leaflet of the plasma membrane mediates the specific recruitment of peripheral proteins such as RAS small GTPases (Sakuragi and Nagata 2023; Leventis and Grinstein 2010).

**Fig. 1:**
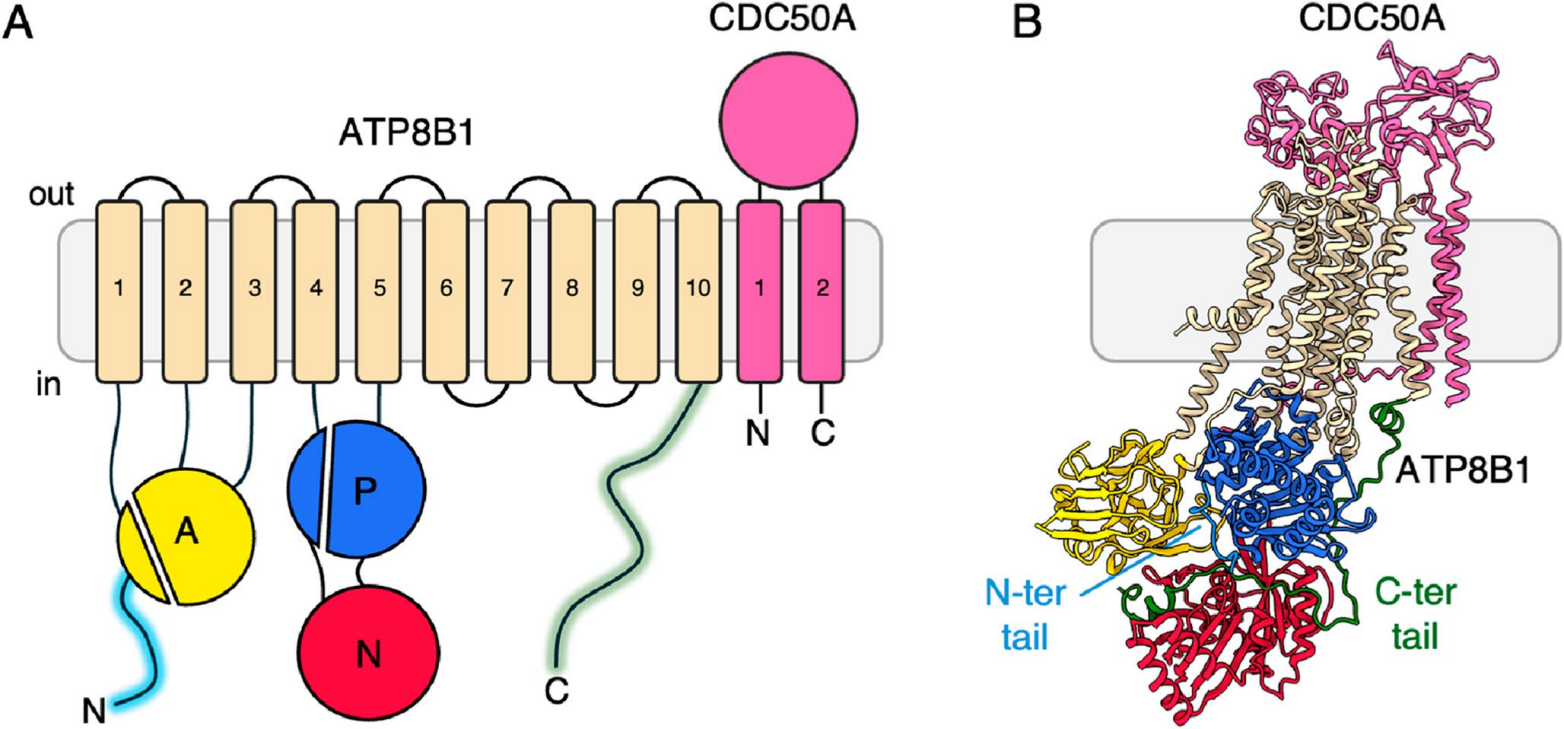
Overall architecture of the ATP8B1-CDC50A flippase complex. (**A**) Topological diagram of the flippase. Color code: the cytosolic A-, N-, and P-domains of ATP8B1 are colored yellow, red, and blue, respectively. The transmembrane domain of ATP8B1 is shown in wheat. The N- and C-terminal tails of ATP8B1 are colored light blue and green, respectively. CDC50A is colored pink. (**B**) Structure of the autoinhibited complex in the E2P_autoinhibited_ conformation (PDB: 7PY4, Dieudonné et al. 2022), with the N- and C-terminal tails bound to the cytosolic domains of ATP8B1. Same color code as in panel A.

P4-ATPases, also known as flippases, participate in the establishment and maintenance of this asymmetry by transporting specific phospholipids from the exoplasmic to the cytosolic leaflet of membranes, in an ATP-dependent manner (Sakuragi and Nagata 2023). P4-ATPases are part of the P-type ATPase superfamily and therefore share a common architecture, with an α-helical transmembrane domain containing the lipid transport site, connected to three cytosolic domains: the nucleotide-binding (N) domain, which binds ATP; the phosphorylation (P) domain, which contains a conserved aspartate residue that is transiently phosphorylated during the transport cycle; and the actuator (A) domain, responsible for transporter dephosphorylation and return of the enzyme to its initial state (Fig. 1) (Palmgren 2023; Stock et al. 2023). In addition, most P4-ATPases form a binary complex with a subunit from the CDC50 family, which mediates their proper function and localization (Bryde et al. 2010). The structural details of the lipid transport cycle of P4-type ATPases have been extensively studied by cryoEM and X-ray crystallography and follow the Post-Albers model, with two main phases: the E1 phase, in which the P4-ATPase undergoes phosphorylation in a lipid-independent manner, and the E2 phase, in which lipid binding and occlusion within the protein trigger dephosphorylation of the conserved catalytic aspartate in the P-domain by an invariant glutamate of the A-domain (Sai and Lee 2024; Duan and Li 2024).

Several P4-ATPases have been shown to be tightly regulated by their N- and/or C-terminal extensions. In particular, the yeast flippase Drs2-Cdc50 and the human flippases ATP8A2-CDC50A and ATP8B1-CDC50A have been reported to be autoinhibited through their terminal extensions (Zhou et al. 2013; Azouaoui et al. 2017; Matsell et al. 2025; Dieudonné et al. 2022). CryoEM structures of the Drs2-Cdc50 and ATP8B1-CDC50A complexes in both autoinhibited and active conformations revealed that the C-terminal tail of these P4-ATPases can establish extensive interactions with the three cytosolic domains of the enzyme after ATP hydrolysis, thereby locking the transporter in E2P autoinhibited conformations (Timcenko et al. 2019, 2021; Cheng et al. 2022; Dieudonné et al. 2022, 2023). In this state, the P4-ATPase is unable to occlude its substrate lipid, a step that is required for lipid translocation. In ATP8B1, this regulatory mechanism is further reinforced by the N-terminal tail, which inserts between the A- and P-domains once the C-terminal tail is engaged, further restricting the cytosolic domain rearrangements required to proceed through the catalytic lipid transport cycle (Fig. 1B) (Dieudonné et al. 2022). Mutations or deletion of this C-terminal tail region in ATP8A2 or Drs2 have been shown to have a drastic effect on protein expression level, likely due to protein instability (Azouaoui et al. 2017; Matsell et al. 2025).

Inherited mutations in P4-ATPases have been associated with various human diseases (Shin and Takatsu 2025). Well-documented examples include progressive familial intrahepatic cholestasis (PFIC) type 1 and the less severe benign recurrent intrahepatic cholestasis (BRIC1), which both result directly from mutations in ATP8B1 (Klomp et al. 2004). While previous studies have investigated ATP8B1 lipid transport specificity and function (Muranaka et al. 2024; Dieudonné et al. 2023), very few studies have explored the regulatory mechanisms of ATP8B1, in particular the role of the different regions of its C-terminal tail in the autoinhibition process.

Previously, our group used purified ATP8B1-CDC50A lacking its C-terminal tail to perform trans-inhibition assays with synthetic peptides to investigate the role of the N- and C-terminal tails of ATP8B1, as well as the putative role of unknown kinase(s) in the activation mechanism of ATP8B1 by phosphorylation of S1223 (Dieudonné et al. 2022). Here, we used a similar approach with peptides corresponding to fragments of the ATP8B1 C-terminal tail to gain further insight into the role of the different sections of this autoinhibitory region of the flippase. This approach allowed us to define the contribution of specific segments, providing a clearer description of the autoinhibition mechanism of ATP8B1 and, more broadly, of other flippases regulated in a similar manner.

## Methods

### ATP8B1Peptides

All peptides presented is this study were synthetized by Biomatik Company (Canada) with > 90% purity grade, trifluoroacetic acid free. The lyophilized peptides were resuspended in buffer A (50 mM MOPS-Tris pH 7, 100 mM KCl), aliquoted and immediately stored at -20 °C. Peptide aliquots were thawed and refrozen between one and six times without noticeable effect on their inhibition properties.

### Heterologous Co-Expression and Purification of the ATP8B1-CDC50A Complex

**Fig. 2:**
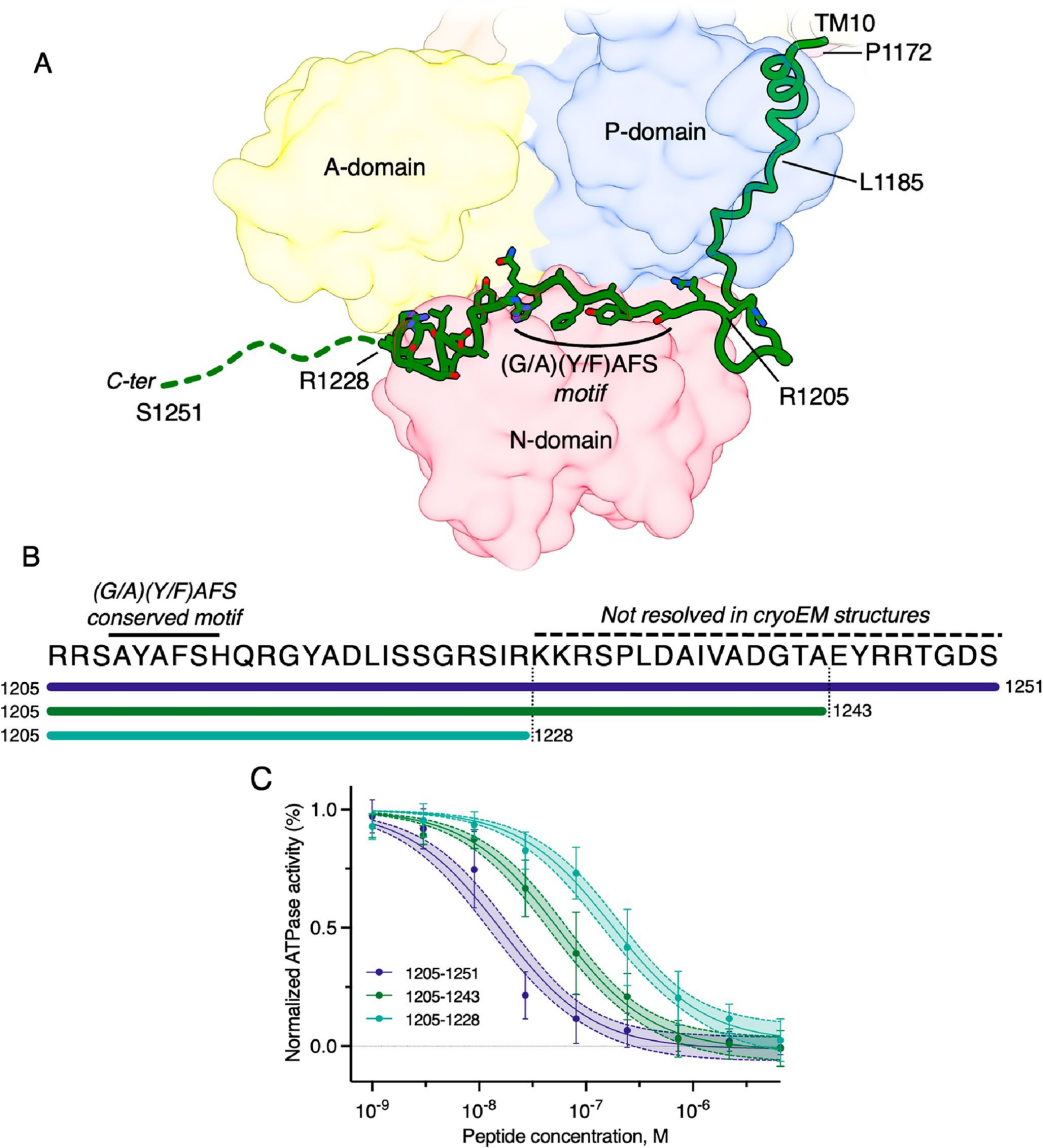
The distal disordered region of the C-terminal tail of ATP8B1 contributes to the autoinhibition mechanism. (**A**) Detailed view of the different parts of the C-terminal tail of ATP8B1 interacting with the cytosolic domains of ATP8B1 in the E2P_autoinhibited_ conformation. P1172 is the first residue after TM10. L1185 is the last residue of ATP8B1 after 3C cleavage of the construct used in this study. R1205 is the first residue of the C-terminal tail mimicking peptides used in this study. R1228 is the last residue of ATP8B1 observed experimentally by cryoEM. S1251 is the last residue of the ATP8B1 full-length sequence. (PDB: 7YP4, Dieudonné et al. 2022). Color code as in Fig. 1. For clarity purposes, the N-terminal tail was omitted. (**B**) Schematic representation of the 1205-1251, 1205-1243 and 1205-1228 peptide sequences mimicking part of the C-terminal tail of ATP8B1. (C) Inhibition of ATP8B1 ATPase activity by increasing concentrations of the corresponding peptides, 1205-1251 (purple), 1205-1243 (green), 1205-1228 (light green). Data were fitted using a dose-response nonlinear regression curve, and the shaded area represents the 99% confidence interval of the fit. Error bars indicate the standard deviation of 3 technical replicates from 3 biological replicates (9 measurements in total). Refer to Table 1 for statistical analysis.

Yeast cultures, recombinant protein expression and membrane preparation were performed as described previously (Azouaoui et al. 2016; Dieudonné et al. 2022, 2023). Purification of C-terminal cleavable hATP8B1(HRV 3C protease L1185)-hCDC50A complex was performed as described previously (Dieudonné et al. 2023). In short, the *Saccharomyces cerevisiae* W303.1b/Δpep4 (MATα, leu2-3, his3-11, ura3-1, ade2-1, Δpep4, can^r^, cir^+^) yeast strain was transformed with the co-expression vector pYeDP60 containing the human cDNA sequences of full-length hATP8B1 (with an HRV 3C protease site inserted at L1185) and hCDC50A. In this construct, ATP8B1 is N-terminally tagged with a TEV-cleavable BAD (biotin acceptor domain), and CDC50A is N-terminally tagged with a decahistidine tag. Transformed yeast were cultured in rich medium before induction of ATP8B1 and CDC50A overexpression by the addition of galactose to the medium. Harvested yeast cells were lysed using glass beads, and membrane fractions were isolated by ultracentrifugation at 120,000 xg for 1 to 2 h. Yeast membranes were then solubilized using n-Dodecyl-β-D-maltopyranoside (DDM) and cholesterol hemisuccinate (CHS) in buffer A (50 mM MOPS-Tris at pH 7, 100 mM KCl, 1 mM DTT, and 5 mM MgCl_2_) supplemented with 20% glycerol (w/v) and proteases inhibitors and the ATP8B1-CDC50A complex was affinity-purified using streptavidin-Sepharose resin. After washing with buffer A supplemented with 20% glycerol (w/v), DDM (0.5 mg.mL⁻¹) and CHS (0.1 mg.mL⁻¹), the complex was eluted with TEV protease, concentrated, and the C-terminal tail of ATP8B1 was cleaved at L1185 (Fig. 2A) by incubation with 3C protease in buffer A supplemented with 20% glycerol (w/v), DDM (0.5 mg.mL⁻¹) and CHS (0.1 mg.mL⁻¹). The complex was then further purified by size-exclusion chromatography on a Superose 6 Increase 10/300 column equilibrated with buffer A supplemented with DDM (0.5 mg.mL⁻¹) and CHS (0.1 mg.mL⁻¹) to remove the TEV and 3C proteases as well as the cleaved C-terminal tail. The final protein concentration of three independent biological triplicates was determined by loading duplicate samples from each purification on a Coomassie blue-stained SDS-PAGE, with purified Drs2-Cdc50 complex of known amount (determined by absorbance at 280 nm) (Fig S1).

### ATPase Activity Assay

To estimate the effect of treating C-terminally truncated ATP8B1 (Δ1185)-CDC50A complex with different peptides, ATP hydrolysis was measured using an enzyme-coupled assay (Sehgal et al. 2016). To measure the ATPase activity of C-terminally truncated ATP8B1 (Δ1185), samples were prepared containing 0.75 µg.mL⁻¹ ATP8B1-CDC50A, 0.15 mg.mL⁻¹ PtdEth (1-Palmitoyl-2-oleoylphosphatidylethanolamine), 0.025 mg.mL⁻¹ PI(3,4,5)P_3_ (D-myo-Phosphatidylinositol 3,4,5-trisphosphate diC16), 0.04 mg.mL⁻¹ PK (Pyruvate Kinase), 0.1 mg.mL⁻¹ LDH (Lactate Dehydrogenase), 1 mM PEP (Phosphoenolpyruvate), 1 mg.mL⁻¹ DDM, and 0.2 mg.mL⁻¹ CHS in buffer A. To allow proper lipid diffusion within flippase-containing detergent micelles, samples were incubated for 1 h 30 min at 4 °C prior to the addition of 165 µM NADH. ATPase activity assays were performed in 96-well plates, with 350 µL sample per well. The decrease in absorbance at 340 nm was monitored for 8 min per run at 37 °C using a SpectraMax^®^ i3 microplate reader in kinetic mode. After an initial run for background measurement, Mg⋅ATP was added to a final concentration of 1 mM to measure ATPase activity prior to peptide addition. This was followed by up to three additional runs with increasing peptide concentrations.

To assess peptide effects on ATPase activity, the activity measured in each run was normalized to the activity of the same sample before peptide addition, after background correction. Each peptide was tested at nine different concentrations ranging from 1 nM to 6.6 µM. Technical triplicates were performed for each concentration and for three independent biological replicates of purified C-terminally truncated ATP8B1 (Δ1185)-CDC50A, resulting in a total of nine measurements per peptide concentration.

### Statistics

IC_50_ of each peptide was estimated, when possible, from the 9 replicates of 9 different peptide concentrations. For each peptide, dose-response nonlinear regression curve fits, as well as the associated 99% confidence band, were generated using GraphPad Prism software based on the associated covariance matrix. For the non-linear fit, the top was constrained to the normalized activity of 1. The non-linear fits are shown in solid, and the 99% confidence bands are shown as transparent bands enclosed in dashed lines.

### Structure Analysis

Molecular graphics and analysis performed with UCSF ChimeraX, developed by the Resource for Biocomputing, Visualization, and Informatics at the University of California, San Francisco, with support from National Institutes of Health R01-GM129325 and the Office of Cyber Infrastructure and Computational Biology, National Institute of Allergy and Infectious Diseases (Pettersen et al., 2021).

## Results and Discussion

### The Distal Disordered Region of the C-terminal Tail of ATP8B1 Contributes to the Autoinhibition Mechanism

To gain insight into the structural basis of ATP8B1 autoinhibition mediated by its C-terminal region, we designed a series of peptides corresponding to different segments of the ATP8B1 C-terminal tail, named according to their position within ATP8B1 (Fig. 2A-B). We then performed trans-inhibition assays of the ATPase activity of C-terminally truncated ATP8B1 (Δ1185) in detergent in the presence of the lipid substrate PtdEth and the activating lipid phosphatidylinositol trisphosphate (PI(3,4,5)P₃), and evaluated their inhibitory properties by determining IC_50_ values (Table 1). As previously observed, the peptide spanning residues 1205-1251, efficiently inhibited ATP8B1 ATPase activity, with an IC_50_ of 16 nM (Fig. 2C; Table 1) (Dieudonné et al. 2022). Truncation of the last eight residues (1205-1243) moderately reduced inhibitory potency, yielding an IC_50_ of 56 nM (Fig. 2C; Table 1). Furthermore, removal of the region corresponding to the disordered segment of the C-terminal tail unresolved in ATP8B1 cryoEM structures (1205-1228) resulted in an approximately tenfold increase in IC_50_ to 175 nM, relative to full-length peptide 1205-1251. These results indicate that the disordered portion of the ATP8B1 C-terminal tail contributes to autoinhibition, likely through transient interactions with the cytosolic domains.

Interestingly, the mammalian flippases ATP8A1 and ATP8A2 appear to be less strongly autoinhibited than ATP8B1, as suggested by the detectable ATPase activity of their full-length proteins, in contrast to ATP8B1 and the yeast homolog Drs2 (Coleman et al. 2012; Hiraizumi et al. 2019; Matsell et al., 2025; Azouaoui et al. 2016; Dieudonné et al. 2022). In line with our results, one possible explanation is that this difference relates to additional interactions involving the longer C-terminal tails of ATP8B1 and Drs2, whereas ATP8A1 and ATP8A2 possess shorter C-terminal tails with smaller disordered distal segments (Fig S2A).

## The E219-R1228 Interaction is Critical for ATP8B1 Autoinhibition

**Fig. 3:**
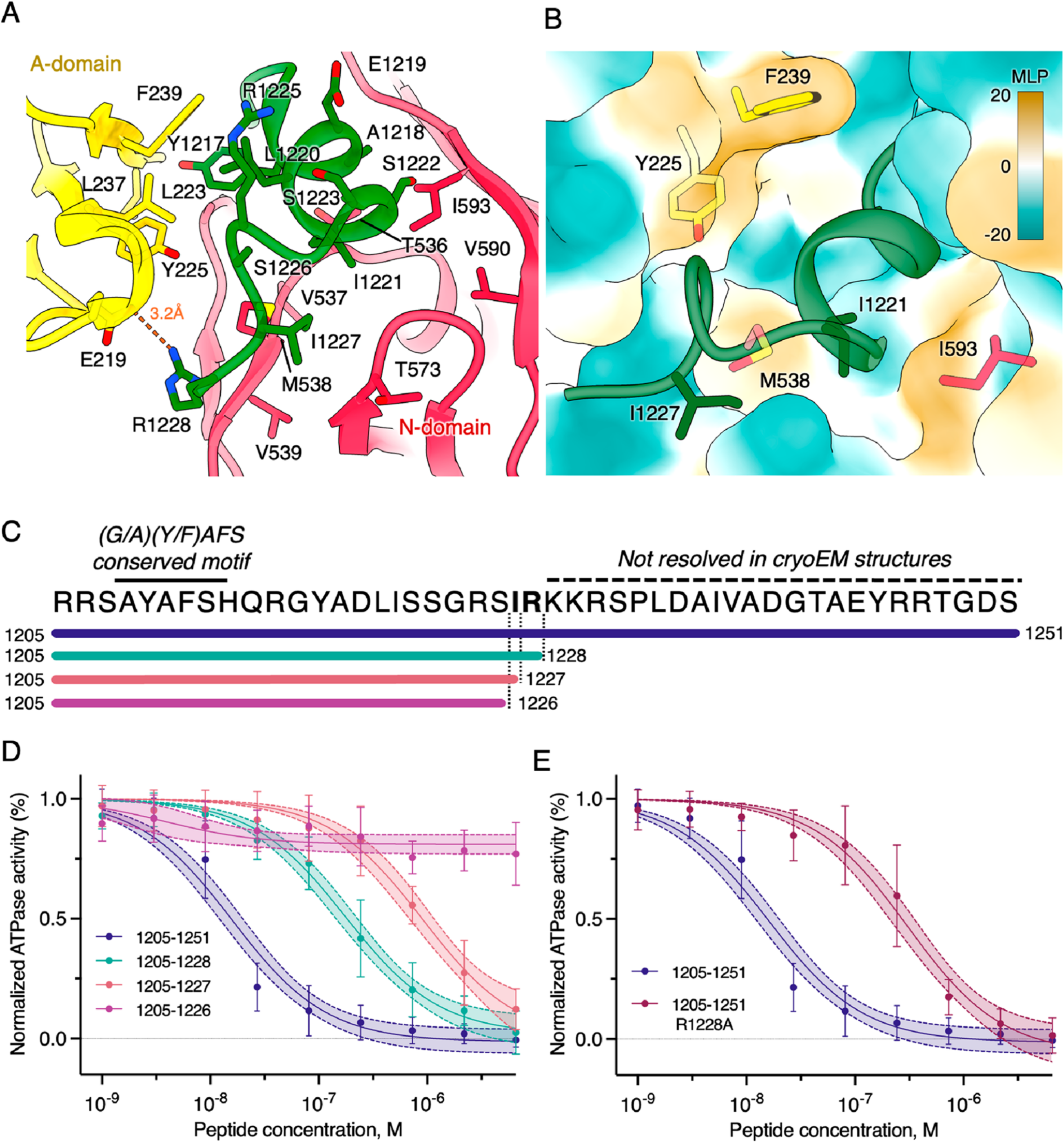
The E219-R1228 salt bridge plays a critical role in the autoinhibition mechanism. (**A**) Close-up view of the interactions of the 1217-1228 region of the C-terminal tail of ATP8B1 with the A- and N-domains in the E2P autoinhibited conformation (PDB: 7YP4, Dieudonné et al. 2022). Color code: the A-, N-domains of ATP8B1 are colored yellow and red, respectively. The C-terminal tail of ATP8B1 is colored in green. The salt bridge between E219 and R1228 is indicated by a dashed line. (**B**) Close-up view of the van der Waals interactions between the A- and N-domain mediated by I1221 and I1227. A- and N-domains of ATP8B1 are shown as surface and colored by molecular lipophilicity potential (MLP) calculated in ChimeraX, with the fauchere method (Pettersen et al. 2021). (**C**) Schematic representation of the 1205-1251, 1205-1228, 1205-1227, and 1205-1226 peptide sequences mimicking part of the C-terminal tail of ATP8B1. (**D**-**E**) Inhibition of ATP8B1 ATPase activity by increasing concentrations of the corresponding peptides, 1205-1251 (purple), 1205–1228 (light green), 1205-1227 (red), 1205-1226 (pink), and 1205-1251 R1228A (plum). Data were fitted using a dose-response nonlinear regression curve, and the shaded area represents the 99% confidence interval of the fit. Error bars indicate the standard deviation of 3 technical replicates from 3 biological replicates (9 measurements in total). Refer to Table 1 for statistical analysis

The most distal region of the ATP8B1 C-terminus observed in the cryoEM structures interacts directly with the A-domain through a salt bridge (E219-R1228) (Fig. 3A) and with the A- and N-domain through van-der-Waals interactions in a hydrophobic patch formed by Y225, F239, M538, I593, I1221 and I1227 (Fig. 3B). Hence, we evaluated the inhibitory properties of shorter peptides lacking either R1228 (1205-1227) or R1228 and I1227 (1205-1226) (Fig. 3C). Removal of R1228 resulted in a fivefold increase in IC_50_, from 175 nM for peptide 1205-1228 to 907 nM for peptide 1205-1227 while the additional removal of I1227 results in an almost complete loss of the inhibition properties of the corresponding 1205-1226 peptide (Fig. 3D; Table 1).

To further assess the contribution of the E219-R1228 interaction, we tested the full-length C-terminal peptide in which R1228 was substituted with alanine (1205-1251 R1228A). This mutant peptide exhibited a 19-fold increase in IC_50_ (305 nM) compared to the WT 1205-1251 peptide, indicating a major contribution of this residue to the autoinhibition (Fig. 3E). However, despite the reduced potency of the R1228A mutant peptide, it retained a stronger inhibitory activity than peptide 1205-1227, with an IC_50_ of 307 nM versus 907 nM, respectively, suggesting that residues located C-terminally to R1228 may partially compensate for the loss of this interaction. In particular, K1229, K1230, and R1231 could potentially engage in electrostatic interactions with E219, consistent with the apparent flexibility of this region of the C-terminal tail.

Interestingly, structural alignment of the C-terminal regions of ATP8B1, ATP8A1, and Drs2 shows that R1228 in ATP8B1 is not conserved (Fig S3). Instead, in ATP8A1 and Drs2, which exhibit a higher degree of sequence and structural conservation with each other, the interaction with the conserved glutamate in the A-domain (E219 in ATP8B1) appears to be mediated by a hydrogen bond involving a conserved tyrosine residue (Y1292 and Y1139 for Drs2 and ATP8A1, respectively) (Figure S3A). However, it is worth noting that E219 is also conserved among P4-ATPases, including those not known to be autoinhibited (Figure S2B).

**Fig. 4:**
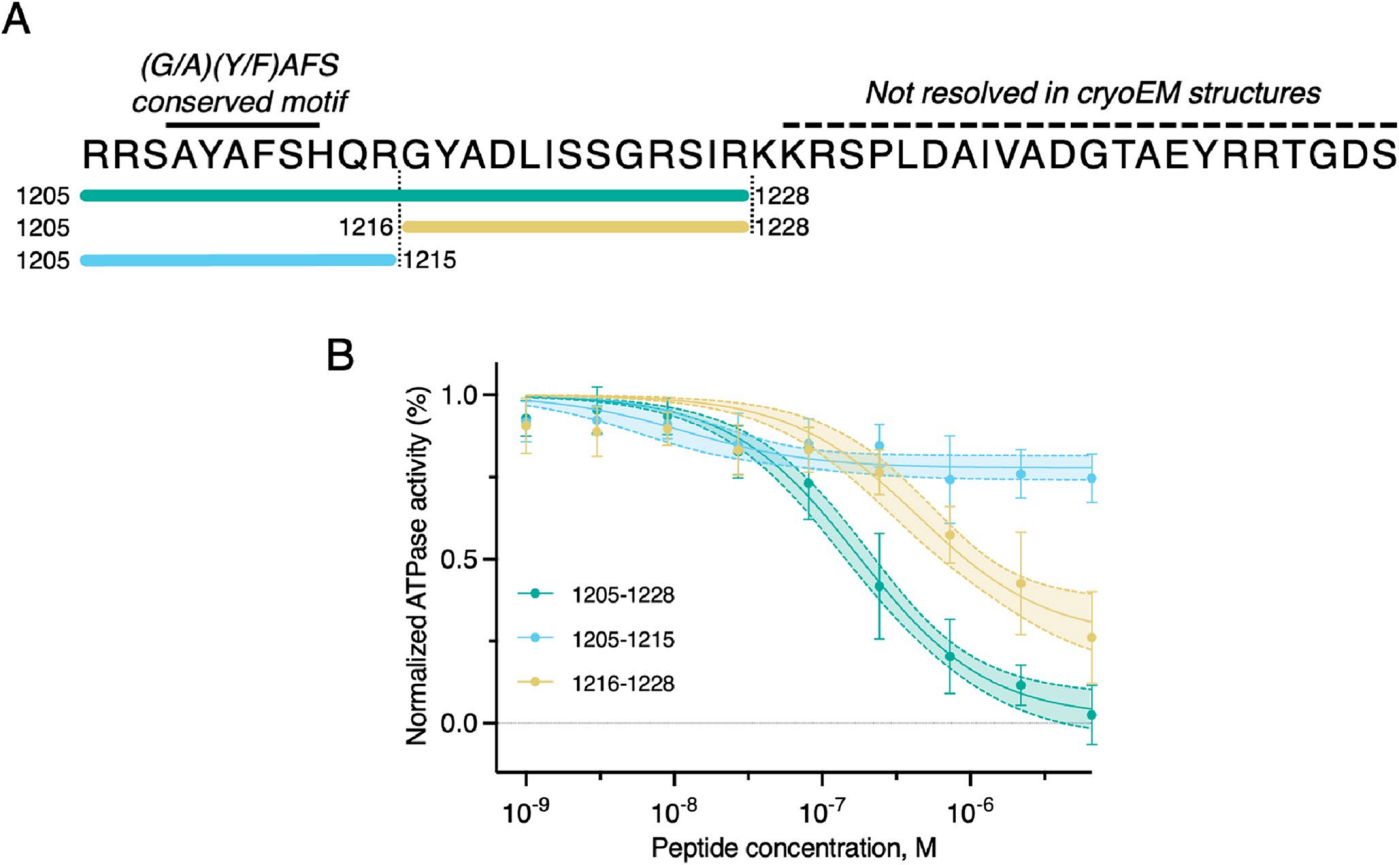
The 1216-1228 fragment is sufficient to inhibit ATP8B1 ATPase activity. (**A**) Schematic rep-resentation of the 1205-1228, 1216-1228, and 1205-1215 peptide sequences mimicking part of the C-terminal tail of ATP8B1. (**B**) Inhibition of ATP8B1 ATPase activity by increasing concentrations of the corresponding peptides, 1205-1228 (light green), 1205-1215 (light blue) and 1216-1228 (gold). Data were fitted using a dose-response nonlinear regression curve, and the shaded area represents the 99% confidence interval of the fit. Error bars indicate the stand-ard deviation of 3 technical replicates from 3 biological replicates (9 measurements in total). Refer to Table 1 for statistical analysis.

### The 1216-1228 C-Terminal Region Bridging the A- and N- Domains is Sufficient for Autoinhibition

Finally, we wanted to investigate the role of the highly conserved (G/A)(Y/F)AFS motif, known to interact with the ATP binding site of the N-domain in the E2P_autoinhibited_ conformation. To this end, we designed two peptides: one spanning residues 1205-1215, containing the conserved motif along with additional polar and positively charged residues to ensure peptide solubility, and a second peptide corresponding to residues 1216-1228, corresponding to the region of the C-terminal tail of ATP8B1 intercalated in between the N- and A-domains (Fig. 4A).

The 1205-1215 peptide did not inhibit ATPase activity, whereas peptide 1216-1228 retained inhibitory properties with an IC_50_ of 445 nM. Although the isolated (G/A)(Y/F)AFS conserved motif (1205-1215) was not sufficient to directly inhibit ATPase activity, its presence markedly enhanced inhibition in the context of the longer peptide (1205-1228) (Fig. 4B; Table 1). Consistently, peptide 1205-1228 exhibited a substantially lower IC_50_ of 175 nM, approximately two-fold lower than that of peptide 1216-1228. Together, these results indicate that the conserved (G/A)(Y/F)AFS motif is not sufficient for inhibition on its own but enhances the inhibitory capacity of the adjacent C-terminal region that intercalates the N- and A-domains. These results are also in line with previous work done on the yeast flippase Drs2 where truncation of its C-terminal tail by limited proteolysis was used to relieve the autoinhibition mechanism in vitro (Azouaoui et al. 2017). Indeed, the truncation by limited proteolysis of Drs2 leaves the (G/A)(Y/F)AFS motif while removing the last 64 residues of the Drs2 C-terminal tail, and results in an active flippase consuming ATP in a lipid-dependent manner. Together with our results, this strongly indicates that the (G/A)(Y/F)AFS motif alone has no inhibitory effect.

**Table 1:**
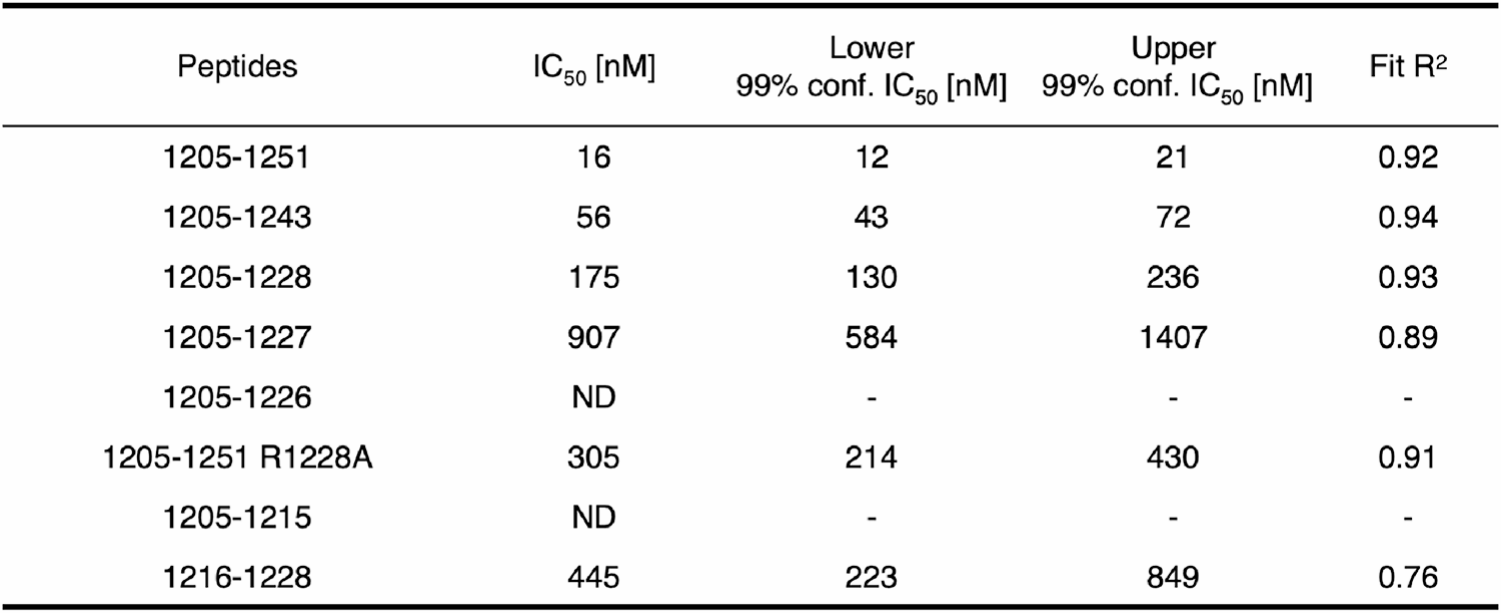
Statistical analysis of the inhibitory properties of the peptides mimicking different regions of the C-terminal tail of ATP8B1

ND: Not determined.

## Conclusion

From a structural perspective, it has been shown that autoinhibited ATP8B1 is locked in an E2P-like conformation, with a possible equilibrium between states with an open or closed lipid-binding site (Dieudonné et al. 2023). Based on this observation, we and others have proposed that C-terminal mediated autoinhibition restricts the rotation of the A-domain, a movement that normally results from structural rearrangements of TM1 and TM2 induced by lipid head-group binding prior to its occlusion within the transport site. Here, our data shows that the minimal region of the ATP8B1 C-terminal tail responsible for autoinhibition corresponds to the 1216–1228 segment that most strongly interacts with the A domain, in line with that model.

More importantly, the A-domain rotation that is blocked by the inhibitory peptide represents a conformational transition, which is highly conserved among P-type ATPases and directly linked to the dephosphorylation of the catalytic aspartate in the P-domain during the transport cycle. Given the strong conservation of this mechanistic step across the P-type ATPase superfamily, our results may have broader implications beyond ATP8B1. In particular, the identification of a minimal peptide segment capable of restricting A-domain motion suggests a potential strategy for the design of inhibitory peptides targeting other P-type ATPases. Conversely, a detailed understanding of this autoinhibitory interaction could also guide the rational design of molecules aimed at antagonizing C-terminal-mediated autoinhibition in P4-ATPases, thereby promoting enzyme activation. Finally, although our results provide important insights into the autoinhibition mechanism of ATP8B1 and P4-ATPases more broadly, how this autoinhibition is relieved in vivo remains to be determined.

## Supplementary Information

Below is the link to the electronic supplementary material.

Supplementary Material 1: ATP8B1 3C protease cleavage and quantification. (A) Coomassie-stained SDS-PAGE analysis of the affinity-purified ATP8B1–CDC50A complex before and after 3C protease cleavage to release the C-terminal tail of ATP8B1. (B) Coomassie-stained SDS-PAGE analysis of affini-ty- and size-exclusion-purified truncated ATP8B1-CDC50A resulting from three independent purifications, quantified using a known amount of the Drs2-Cdc50 complex.

Supplementary Material 2: Structural alignment of ATP8B1, ATP8A1 and Drs2 C-terminal tail regions. (A) Structural alignment of the most distal part of the C-terminal tails of ATP8B1 (green), ATP8A1 (light blue), and Drs2 (dark blue) as resolved in their respective cryoEM structures. ATP8B1 (PDB: 7PY4, Dieudonné et al., 2022), ATP8A1 (PDB: 6K7L, Hiraizumi, et al., 2019), and Drs2 (PDB: 6ROH, Timcenko et al., 2019) were aligned based on their N-domains. (B) Corresponding sequence alignment showing the Cα distances measured in ChimeraX. The two hydrophobic residues of the hydrophobic patch are underlined with a *. (C) P4-ATPases C-terminal tail sequence comparison after structural alignment.

Supplementary Material 3: ATP8B1 protease cleavage and quantification. (A) Coomassie-stained SDS-PAGE analysis of the affinity-purified ATP8B1–CDC50A complex before and after 3C protease cleavage to release the C-terminal tail of ATP8B1. (B) Coomassie-stained SDS-PAGE analysis of affini-ty- and size-exclusion-purified truncated ATP8B1-CDC50A resulting from three independent purifications, quantified using a known amount of the Drs2-Cdc50 complex.

## Data Availability

The data used to support the findings of this study are available from the corresponding authors upon request.
