# Supplementary figures and images for: Mechanistic Insights into Autoinhibition of the Human Flippase ATP8B1
Source: J Membr Biol. 2026 Jul 28;259(1):26. doi: 10.1007/s00232-026-00391-6 (PMC13415658; doi:10.1007/s00232-026-00391-6)

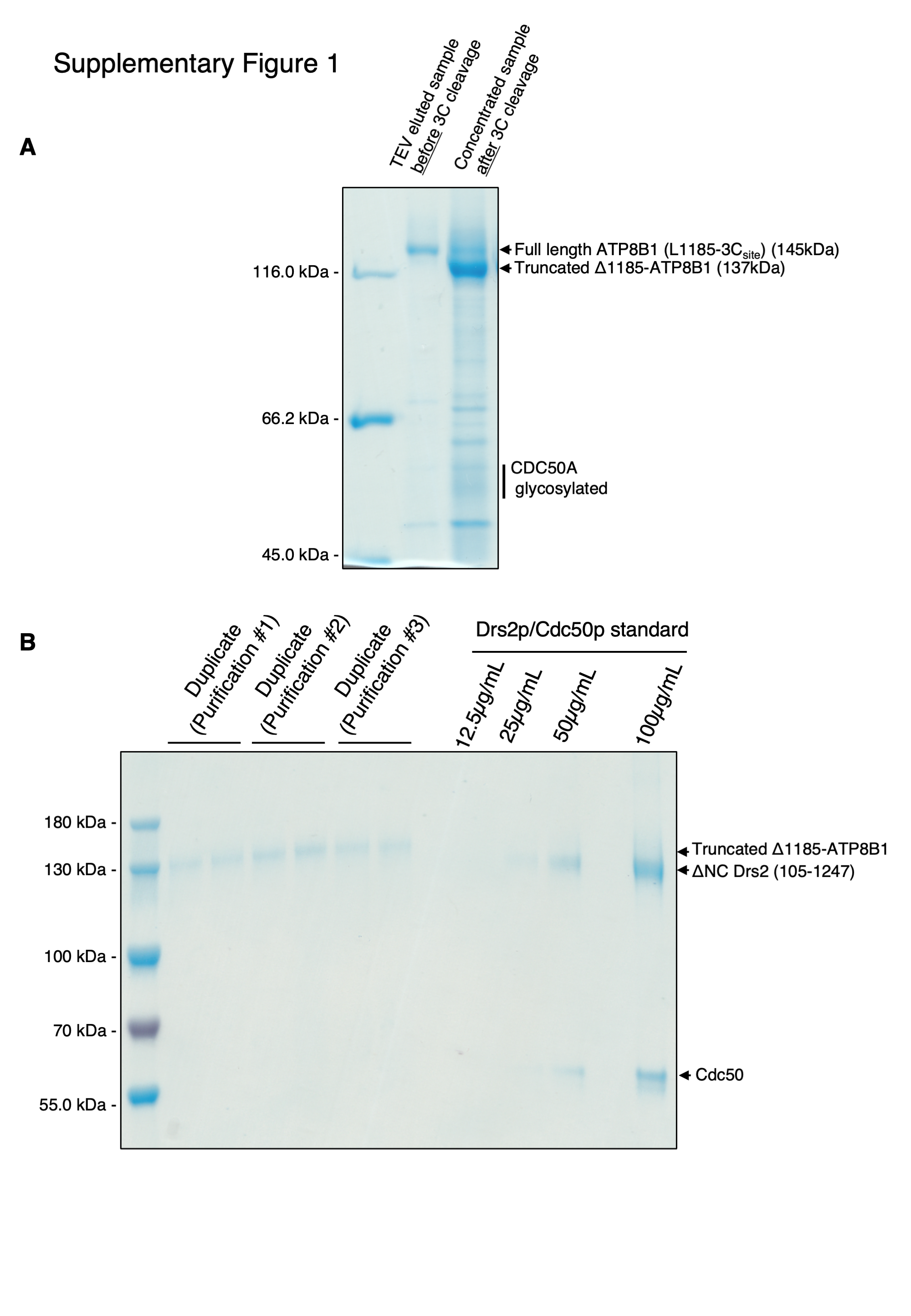

Supplement: Supplementary file 1 — Supplementary Material 1: ATP8B1 3C protease cleavage and quantification. (A) Coomassie-stained SDS-PAGE analysis of the affinity-purified ATP8B1–CDC50A complex before and after 3C protease cleavage to release the C-terminal tail of ATP8B1. (B) Coomassie-stained SDS-PAGE analysis of affini-ty- and size-exclusion-purified truncated ATP8B1-CDC50A resulting from three independent purifications, quantified using a known amount of the Drs2-Cdc50 complex. [file 232_2026_391_MOESM1_ESM.tiff]

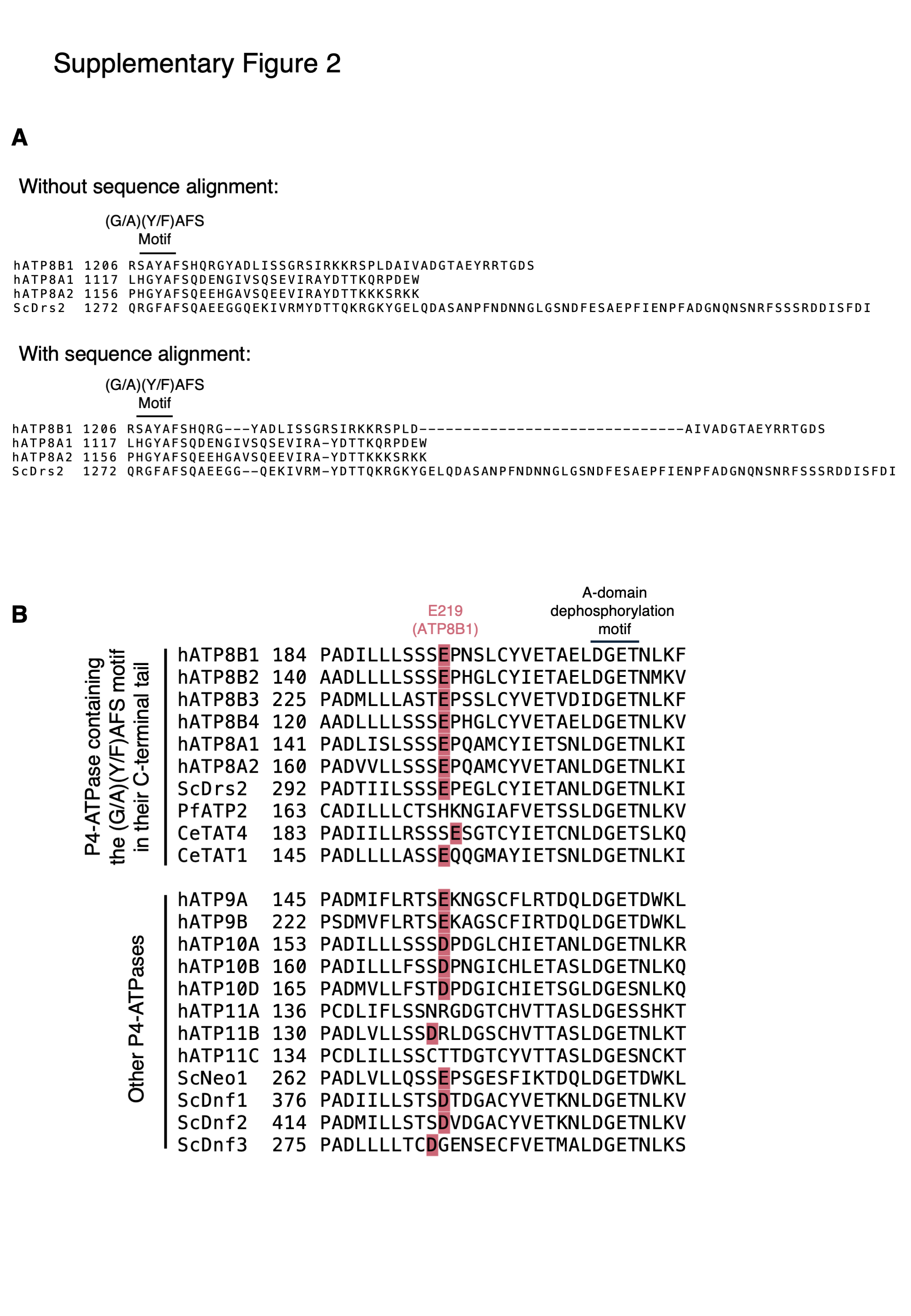

Supplement: Supplementary file 2 — Supplementary Material 2: Structural alignment of ATP8B1, ATP8A1 and Drs2 C-terminal tail regions. (A) Structural alignment of the most distal part of the C-terminal tails of ATP8B1 (green), ATP8A1 (light blue), and Drs2 (dark blue) as resolved in their respective cryoEM structures. ATP8B1 (PDB: 7PY4, Dieudonné et al., 2022), ATP8A1 (PDB: 6K7L, Hiraizumi, et al., 2019), and Drs2 (PDB: 6ROH, Timcenko et al., 2019) were aligned based on their N-domains. (B) Corresponding sequence alignment showing the Cα distances measured in ChimeraX. The two hydrophobic residues of the hydrophobic patch are underlined with a *. (C) P4-ATPases C-terminal tail sequence comparison after structural alignment. [file 232_2026_391_MOESM2_ESM.tiff]

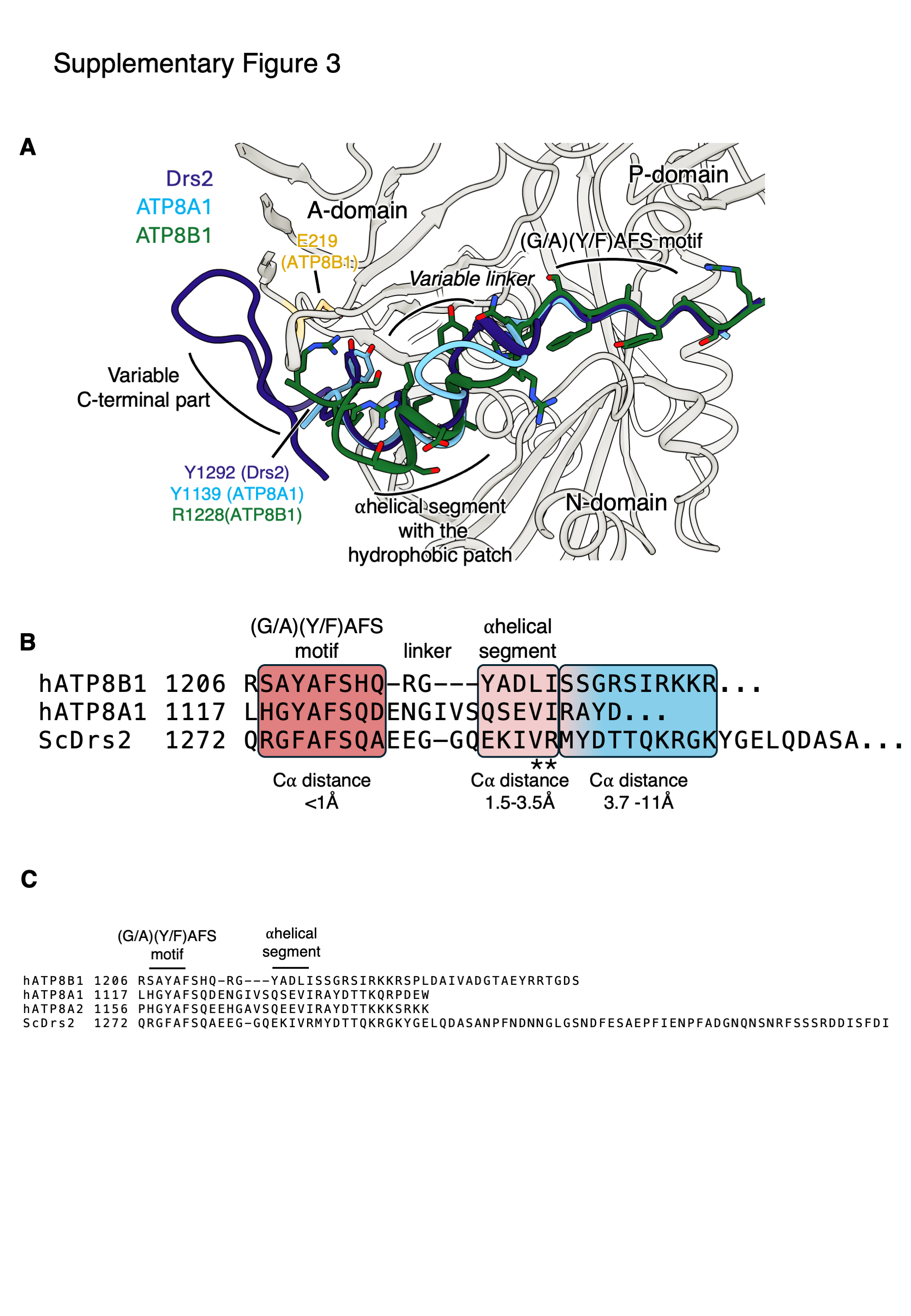

Supplement: Supplementary file 3 — Supplementary Material 3: ATP8B1 protease cleavage and quantification. (A) Coomassie-stained SDS-PAGE analysis of the affinity-purified ATP8B1–CDC50A complex before and after 3C protease cleavage to release the C-terminal tail of ATP8B1. (B) Coomassie-stained SDS-PAGE analysis of affini-ty- and size-exclusion-purified truncated ATP8B1-CDC50A resulting from three independent purifications, quantified using a known amount of the Drs2-Cdc50 complex. [file 232_2026_391_MOESM3_ESM.tiff]
